# A cost-efficient and alternative technique of managing fall armyworm *Spodoptera frugiperda* (J.E. Smith) larvae in maize crop

**DOI:** 10.1038/s41598-022-10982-7

**Published:** 2022-05-05

**Authors:** Ujjawal Kumar Singh Kushwaha

**Affiliations:** National Plant Breeding and Genetics Research Centre, Nepal Agricultural Research Council, Khumaltar, Lalitpur, Nepal

**Keywords:** Plant sciences, Environmental sciences

## Abstract

An experiment was conducted to test the efficacy of grease and emamectin benzoate in a randomized complete block design with five replications to reduce fall armyworm, *Spodoptera frugiperda* (J.E. Smith) larvae load from a maize field in the winter seasons of 2020 and 2021 in Sarlahi, Nepal. Standard agronomic package of practices followed for crop proper growth and development, and plant spacing maintained at 20 × 60  cm^2^ with a plot size of 390 m^2^. The treatments were applied when the maize crop was at knee height and larvae damaged nearly 5–8% of the total plants. Emamectin benzoate sprayed at 0.4 g/liter of water and grease of about 0.15 g applied to the maize whorl or tip of a drooping leaf that touched the soil. A significant reduction in larval infestation was observed after 7-days of treatment applications. Fall armyworm larvae were found dead in the chemical-sprayed plots, but they were absent in the grease-applied fields. No crop damage was observed among the grease-treated plants, which might be due to restrictions in the movement of larvae on the maize crop. The armyworm larvae might get irritated, feel insecure, and move far away from the test plots searching for food materials. Thus, an eco-friendly material like grease can be used as an agroecological method for managing fall armyworm larvae among small-scale land-holding maize farmers.

## Introduction

The fall armyworm, *Spodoptera frugiperda* (J.E. Smith), is a voracious agricultural pest of global economic importance^1^. This polyphagous pest is native to North and South America attacks and feeds on over 350 plant species while easily surviving and reproducing in a variety of tropical and subtropical environment^1–3^. The adult fall armyworm is a well-known sporadic species and can migrate up to 100 km in a single night. Therefore, it has become a fascinating pest in many countries for its proper management^4,5^. Several physical, chemical, and biological control strategies have been tried, but most are unsatisfactory for this destructive pest^6^. Hence, it has created food insecurity mainly among small land-holding farmers in Asia and Africa who grow maize as their main staple food^7,8^. Preventive measures like a quality seed, early planting, field sanitation, conserving the pest’s natural enemy, push–pull technology, and plant diversity could reduce fall armyworm infestation to a large extent^9^.

Maize is the main staple food crop of the peoples of Nepal’s hills, ranking second only to rice in terms of area, production, and productivity^10^. The country and its farmers have been facing many challenges like the timely unavailability of quality seeds, chemical fertilizers, and pesticides, and as a result, national productivity (2.82 t/ha) is less compared to other South Asian countries^11^. Additionally, the maize crop faces a challenge from an invasive pest, the fall armyworm, which causing devastation throughout the country since 2019^9^. Now fall armyworm has become the major pest of maize in Nepal. Small farmers are adversely affected, and management methods are inadequate to cope with the problems^1^. No detailed assessment is available to understand the loss from armyworms across the nation. According to Aguirre et al.^12^, fall armyworm can cause a 30% yield loss in general, whereas Kumela et al.^13^ reported crop damage of up to 32% in Ethiopia and 47.3% in Kenya, with an estimated yield reduction of 0.8 to 1 ton/ha. Similarly, Groote et al.^3^ reported that FAW caused a loss of about one-third of the annual maize production in Kenya, which rated at 1 million tons.

Nepalese farmers mainly use conventional chemical control methods with a combination of insecticides to combat fall armyworm larvae. However, most of the chemicals have not been found satisfactory to eradicate this invasive pest, which might be due to a lack of farmers’ knowledge of insecticides, weak purchasing power with a tendency to select cheaper products, inaccessibility of appropriate and effective products, and a rise in the resistance level of pesticides to this pest^4,13^. Pesticide overuse has had negative effects on soil, water, air, and plant biomass, resulting in long-term environmental degradation, and an increase in cultivation costs^1^. Pest control is difficult due to pesticide resistance^14^, the availability of diverse alternative host plants^15^, adult migratory behavior, and the hide-and-seek nature of larvae^2^. Thus, this study aimed to apply agroecological approaches that have become more relevant to smallholder farmers who lack financial resources to purchase chemical pesticides. Here, glue-like material grease was tested for managing fall armyworm larvae by interrupting their locomotion action over the maize leaf surface. Grease is a solid or semi-solid glue-like water-insoluble material that works as a lubricant in automobile vehicles. It is a non-toxic, degradable, thick petroleum jelly with a specific odor and is highly sticky^16^. Previously, several sticky-like materials (yellow sticky traps) were widely used to control crop insects and pests^17,18^.

## Materials and methods

### Experimental site

This experiment was conducted at Godaita-5 municipality and Dhankaul-1 rural municipality in Sarlahi, Nepal. These two municipalities are located in the plain part of Nepal near the southern Indian border. Sarlahi district lies at the latitude and longitude of N 27° 0′ 30.2724ʺ and E 85° 31′ 12.0864ʺ with an average height of 62 m above the mean sea level. Sarlahi has a humid tropical climate with a mean annual rainfall of 2214 mm. The monthly mean maximum temperature, minimum temperature, rainfall, and humidity data were retrieved from https://www.worldweatheronline.com (Table 1).

**Table 1:** The monthly mean maximum and minimum temperatures with rainfall and humidity data for the winter and summer season maize growing periods of Malangawa Sarlahi (2019/2020 and 2020/2021) and Khumaltar Lalitpur (2021), Nepal.

2019/20	Mean maximum temperature (°C)	Mean minimum temperature (°C)	Average temperature (°C)	Mean rainfall (mm)	Mean humidity (%)
October	31	23	28	12.23	71
November	31	22	28		55
December	25	16	21		55
January	22	14	19	19.6	52
February	25	16	22		56
March	32	22	29		40
**2020/21**					
October	33	26	30	8.86	60
November	29	21	26		45
December	26	17	23		38
January	25	15	21	0.4	48
February	28	16	24		39
March	37	22	32		22
**2021**					
March	28	15	24	371.3	
April	31	18	28		28
May	26	17	23		63
June	26	19	24	965.6	87
July	24	19	22		90
August	23	19	22		92

Experimental site weather data retrieved from https://www.worldweatheronline.com.

### Experimental methods

To manage fall armyworm larvae in the experimental fields, two treatments were tested in a randomized complete block design with five replications in the winter season of 2019/20. Fifteen farmers' fields each measuring 390 m square, were selected. Each area was supposed to be a single plot. Maize hybrid CP 808 was planted with a spacing of 60 × 20 cm^2^ in the third week of October 2019. A farmer-based agronomic package of practices was followed and the nationally recommended dose of fertilizer (NPK@120:60:40 kg/ha) was applied for maize’s proper growth and development^10^. The three treatments used in the experiment were grease, emamectin benzoate, and the third treatment remained as a control.

**Figure 1:**
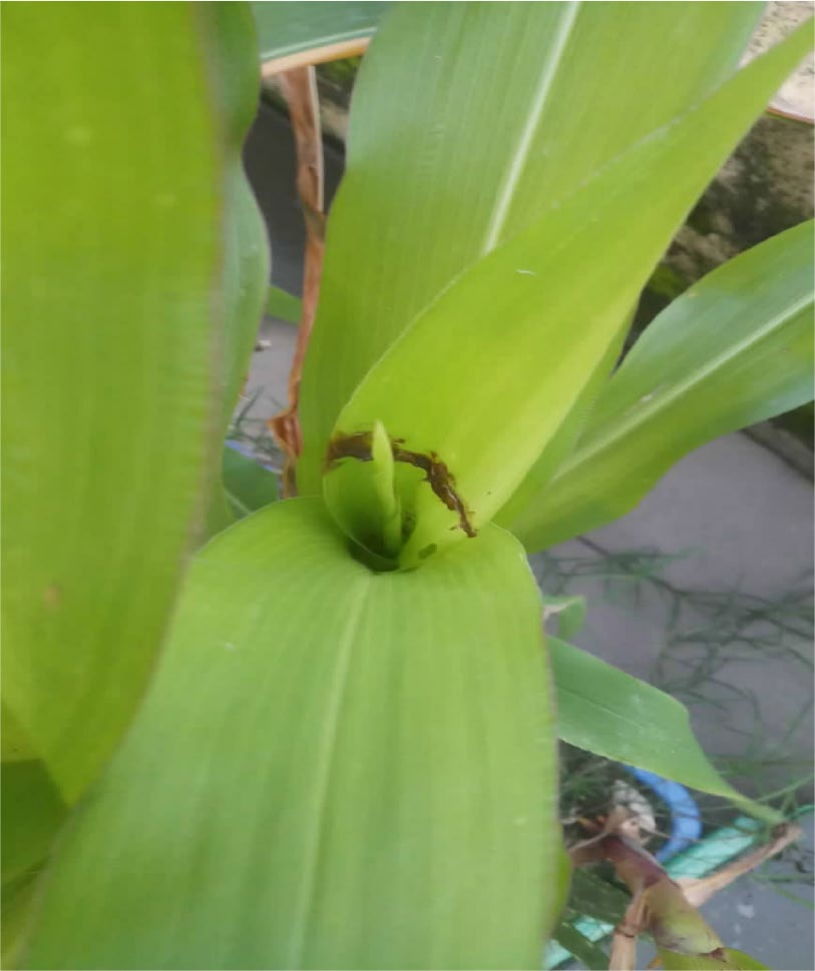
Shows the application of grease to a maize new leaf around the whorl.

Grease applied at 0.15 g near the maize whorl or leaf near the maize whorl. For the grease test (trade name: MP-3 Grease, Nepal Multilube System Ind. Birganj, Nepal), each plot was demarcated diagonally, with only the plant whorl that belongs on the diagonal line plus the infested whorl per plot included. Grease was applied as if maize were at the knee height stage, and the plants had a larval infestation of 5–8%. It approached only once in the maize crop whole growth period (Fig. 1). Similarly, emamectin benzoate (5% SG) (Trade name: EM-1, Dhanuka Agritech Limited, India) was sprayed at 0.4 g/liter into the respective fields. The control plot was left as well, without applying any treatments.

To validate the first year’s experimental results, six farmer’s fields of 333 m square were selected, and a randomized complete block design was used to study the trial. Each of the two field plots was fixed for grease and emamectin benzoate, respectively, followed by control, and planting done as a winter season maize in 2020/21. The maize hybrid variety P3522 was intercropped with potato var. Rajendra-1, sown in the first week of November 2020. With severe fall armyworm larvae damage (50% of the total crop) on standing maize, the plots were demarcated diagonally, and the plants along the diagonal line along with the infested whorl per plot had a small amount (0.10-0.15g) of grease applied to the portion of the maize lower leaf that touched the soil.

### FAW larvae artificial infestation and field results validation

To validate the output of field results, the maize variety Mankamana-4 was grown in 20 pots under non-controlled conditions at Khumaltar, Lalitpur, Nepal in the summer season of 2021. The plants were expanded in the cage and artificially infested with first and third instar fall armyworm larvae four times during the whole growth period. The first artificial infestation (3–4 larvae per plant) was made when the maize crop was at the two-leaf stage, the second and third at knee height stage with a 15-day interval, and the fourth at the crop heading stage. A small quantity (0.20 g) of grease registered on the maize whorl after two days of artificial infestation. In knee-height stage 2, the pots were split into two plots, each consisting of 10 plants, where one served as a grease treatment and the other as a control (Table 4). The plots were half-covered with a plastic net. In the earlier growth stage, only grease treatment was done. After 16 days of infestation, the larva found in the control plot was removed from the infested plants. The potted plants were kept under the investigator’s strict supervision.

### Data collection and analysis

Each plot was monitored by a group of five farmers before and after the treatment applications. The farmers’ fields were monitored twice at 7-day intervals until the fields were free of the devastating fall armyworm larvae. The number of affected and non-affected plants was counted before and after the treatment applications. The affected plants were counted in each plot, and the unaffected plants were estimated by reducing the affected plants from the total number of plants in each plot, respectively. The plant that showed fall armyworm larvae infestation was considered an affected or damaged plant and the non-infested plant was taken as non-affected or undamaged. Fall armyworm larvae infestation and its damage on maize plants were estimated using a simplified day-independent rating scale (“Davis’ 0 to 9 whorl and furl damage scale”)^12,19^. For damage scoring, each field was demarcated diagonally and alternated 10 plants’ leaves, with their whorl was chosen from the center of the plot for rating purposes. The grain yield of each plot was considered based on the farmer’s interviews that took place after the crop’s post-harvest. The recorded data were entered into MS Excel and analyzed through the MSTAT software program (http://web.archive.org/web/20111012082739/http://www.msu.edu/~freed/disks.htm).

### Ethics statement

I confirm that the experimental research and field studies followed all ethical guidelines of the Nepal Agricultural Research Council and the government of Nepal. Informed consent was obtained from all participants.

## Results

### Identification of grease as a potential fall armyworm larvae deterrent

A significant reduction in fall armyworm larval infestation was observed in the grease-treated plots after 7-days of treatment application. However, the field crops were free from the larvae after 14 days. The maize crop had new leaves and whorls without any damage. In the field plots, larvae were not found either the dead or alive form. Only 22 plants were damaged out of 2775 in just 7-days, and no damaged plants were observed after 14 days. The larvae had not been found ingesting grease applied to maize leaves during the treatment period. Besides, emamectin benzoate also showed promising results. However, the fall armyworm larvae were still found in the maize whorl (2 plants affected) after 7-days of spray. Most of the larvae were found dead with this chemical treatment. But the control plot showed heavy maize infestation and nearly 40% of plants affected after 14 days, though the damage rating score was less (1.88) than the grease and emamectin benzoate plots in 2019/20 (Table 2).

**Table 2:** Analysis of variance shows average grain yield, damage rating score, and number of affected and unaffected maize plants after the application of grease and emamectin benzoate in experimental plots to control fall armyworm larvae in the winter seasons of 2019/2020.

SN	Treatment	Average number of plants before treatment		Average number of plants after treatment application				Average yield (kg/ha)	Average damage rating score
		Affected	Unaffected	Affected at 7 days	Unaffected at 7 days	Affected at 14 days	Unaffected at 14 days		
1	Grease	217	2558	22	2753	1	2774	5370	2.06
2	Emamectin benzoate	232	2543	2	2773	0	2775	5260	2.02
3	Control	230	2545	411	2364	1120	1655	3880	1.88
Grand mean		226	2549	145	2630	374	2401	4837	1.98
Coefficient of variation (%)		36.07	3.2	50.41	2.78	39.29	6.12	4.61	34.77
Standard Error		18.29	18.29	53.48	53.48	145.38	145.38	196.67	0.17
p value				0	0	0	0	0	

ANOVA was calculated based on three treatments with five replications. Two number after decimal is reduced to a single digit.

*P* value calculated at 0.05% level of confidence.

The highest yield was obtained in grease-treated plots, followed by emamectin benzoate plots, and the lowest in control plots. The average yield of grease and emamectin benzoate treated plots was 5370 kg/ha and 5260 kg/ha, respectively. The control plot had an average yield of 3880 kg/ha. To deter fall armyworm caterpillars from maize test plots, the majority of farmers (80%) responded that grease-treated plots were more effective than chemical-treated plots.

### Grease interrupts FAW larvae movement

There was severe larval infestation found in maize experimental plots with average damage rating scores ranging from 4.5 to 7 before the application of grease and emamectin benzoate in the winter season of 2020/21. Maize new leaves started to emerge with an attractive whorl after 7-days of treatment when a small amount of grease was applied at the tip of maize drooping leaves touching the ground. No fall armyworm larvae were found either dead or alive after 14 days, and the fields were free from damage up to the crop harvesting stage. Similarly, emamectin benzoate also showed promising results, killing all larvae within 7 days of treatment application. The significant average yield was achieved in grease-treated plots (7256 kg/ha), followed by emamectin benzoate (7160 kg/ha), but control plots had a comparatively lower yield of 4094 kg/ha (Table 3).

**Table 3:** Analysis of variance shows average grain yield, damage rating score, and number of affected and unaffected maize plants after grease and emamectin benzoate applications to control fall armyworm larvae in the winter season of 2020/2021.

SN	Treatment	Average number of plants before treatment		Average number of plants after treatment application				Average yield (kg/ha)	Average damage rating score
		Affected	Unaffected	Affected at 7 days	Unaffected at 7 days	Affected at 14 days	Unaffected at 14 days		
1	Grease	1436	1265	110	2590	0	2700	7256	7
2	Emamectin benzoate	1299	1401	0	2700	0	2700	7160	5.5
3	Control	1162	1538	1722	979	2460	240	4094	4.5
Grand mean		1299	1401	611	2090	820	1880	6170	5.7
Coefficient of variation (%)		22.87	21.2	13.24	3.87	5.08	2.21	4.45	19.06
Standard Error		95.43	95.43	352.83	352.83	518.78	518.78	660.81	0.56
p value				0.0035	0.0035	0.0004	0.0004	0.0115	0

ANOVA was calculated based on three treatments and two replications. Two number after decimal is reduced to a single digit.

*P* value calculated at 0.05% level of confidence.

### Grease manages fall armyworm larvae in artificial infestations

Repeated artificial infestation of fall armyworm larvae during maize different growth stages showed that the third instar larvae tried to move through the grease-based line, and they left the infested plants after 1–2 attempts. Similarly, the first instar larvae fall from the maize plant to the soil surface when they touch the grease applied to the leaves. The FAW caterpillars were also seen as uninterested in moving over grease-applied plants, and instead, these larvae chose new plants on which either grease was not applied or they preferred new sites. Field-based natural and cage-based artificial infestation of fall armyworm larvae resulted in that grease effectively disturbing fall armyworm larval movement. Fall armyworm larvae were not found in the maize plants after 3–4 days of grease application under artificial infestation. The grease did not show any side effects on the maize leaves during the whole growth period. Additionally, the corn-strain larvae have been observed to exhibit unique locomotion behavior where they become very active during the night period and migrate from one plant to the other through maize-drooping leaves in search of food materials for their survival. When grease was applied to the drooping leaves, they changed their route and left the plants. Similarly, FAW larvae also found specific feeding behavior that preferred tender new whorls and their leaves during the maize early growing period and silk and kernel tissues during the maize reproductive stage (Table 4).

**Table 4:** Fall armyworm larval damage rating score and feeding preference of potted maize plants in artificial infestation condition, 2021.

SN	Maize growth stage	Treatment	No. of plants before treatment		No. of plants after treatment application				Larvae feeding preference	Larvae location preference during day	Average grain weight (g/ear)	Average damage rating score
			Damaged	Undamaged	Damaged at 7 days	Undamaged at 7 days	Damaged at 14 days	Undamaged at 14 days				
1	2- leaf stage	Grease	8	12	0	20	0	20	Tender leaves	Soil	–	2
2	Knee stage^1^	Grease	14	6	2	18	0	20	Whorl and new leaves	Whorl	–	5
3 i	Knee stage^2^	Grease	7	3	0	10	0	10	Whorl and new leaves	Whorl	–	3
ii		Control	8	2	10	0	10	0		Whorl	–	6
4 i	Heading stage	Grease	4	6	1	9	0	10	Silk and kernel tissue	Whorl	199	1
ii		Control	6	4	10	0	10	0			141	4

## Discussion

Fall armyworm larvae move from one plant to the next, mainly through the maize-drooping leaves, and consume tender new whorls for their survival. During the night period, they become active and locomote from one plant to the next. When grease applies to the maize whorl or tip of a drooping lower leaf that touches the soil, it sticks to the larvae’s locomotory appendages and restricts their movement. They become disturbed and irritated due to barriers in their path. Thus, they detect danger, feel insecure, and move far away from the test plots to search for food materials. Alternatively, grease might have an irritating and unpleasant smell that respells FAW larvae from the grease-applied plants. Grease was selected for managing fall armyworm larvae because it is water-insoluble and remains highly sticky even after heavy dew or rainfall for a couple of days. Sticky materials are applied widely to control and monitor insects and pests in field crops^18^. Broughton and Harrison^20^ used a yellow sticky trap for monitoring onion thrips (*Thrips tabaci*), whereas Kim et al.^17^ used the sticky-trap to control the density of *Ricania shantungensis* in blueberries. Similarly, specific intercrops (maize + *Desmodium* spp.) are also used to reduce pest infestations by reducing the movement of larvae between the crop plants and thereby reducing larval dispersal^1,4,6,21^. To repel pests, certain chemicals with repellent properties are used, and Wallingford et al.^22^ reported that the odor of 1-octen-3-ol and geosmin treated fruits inhibited the behavior of *Drosophila suzukii* and reduced red raspberry oviposition, thus protecting the fruits.

Emamectin benzoate showed promising results in the field plots, killing most of the fall armyworm larvae. Only those larvae that appeared on the leaf surface or ingested new leaves during the residual period were killed, but some might not be killed because they might hide in the whorl or the soil or move far from the chemical-treated fields. As a result, two sprays at specific day intervals are required to protect the crop from this invasive pest, or else the larval infestation will re-infest the crops. Similar results were reported by Babendreier et al.^7^, who tested insecticide emamectin benzoate against FAW in field conditions and found a significant reduction in larval numbers and crop damage with increased yields in the Upper West and Greater Accra regions of Ghana. In addition, Deshmukh et al.^23^ also reported the highest acute toxicity of emamectin benzoate (5 SG), followed by chlorantraniliprole (18.5 SC) and spinetoram (11.7 SC) in leaf-dip bioassay and field conditions revealed the effectiveness of the insecticides, which was correlated with higher grain yields in comparisons with control. Similar results were reported by Bajracharya et al.^24^, Bansode et al.^25^, and Thumar et al.^26^.

The maximum yield was achieved in grease-treated plots at par with chemical treatment because both plots were free from fall armyworm larvae infestation and had received similar agronomic management. But farmers responded that grease-treated plots had better crop performance and net total return than emamectin benzoate since grease is a cheap material, cost-effective, eco-friendly, degradable in nature, easy to apply at crop any growth stage, and has no hazardous effects on the environment or any other beneficial organisms than the chemical insecticide emamectin benzoate^7,16^. However, the control plot had the lowest yield because the plant leaves were highly damaged by fall armyworm larvae, which interrupted the total photosynthesis of the crop, resulting in a lower yield.

The artificially infested fall armyworm larvae showed a learned response when coming into contact with grease. The larvae did not cross the grease-made borderline and left the plants. Thus, FAW larvae showed learned behavior, which means that they sensed the danger and left the plants to make themselves safe. Similarly, the larvae were reported to prefer tender new leaves and whorls, and a similar result was also reported by Pannuti et al.^27^, who found that the first instar larvae had a strong effect on feeding choice and that they chose silk and kernel during the corn flowering and fruiting stages. Information on larval behavior, including movement and feeding, is important as it helps to design the strategies to manage the pest^27^. Corn earworm (*Helicoverpa zea* Boddie) is also reported to behave similarly to fall armyworm, penetrating the ear and feeding on kernel tissues^28^.

## Conclusion

The fall armyworm is an invasive pest and is widely destructive in nature. Though the chemical control method is effective, it has shown hazardous effects on the total environment. Alternatively, water-insoluble, sticky, and oily materials like grease showed promising results by interrupting larvae locomotion action over the maize leaf surface. This sticky material is cheap, degradable, easy to use, and cost-effective for small land-holding farmers. Hence, it can work as an agroecological method for pest control in maize crops.

## Data Availability

Original data will be provided if needed.

## Code availability

Software applications are mentioned in the methods, and programs are publicly available.
