# Author Correction: A cost-efficient and alternative technique of managing fall armyworm *Spodoptera frugiperda* (J.E. Smith) larvae in maize crop

**DOI:** 10.1038/s41598-022-16484-w

**Published:** 2022-07-25

**Authors:** Ujjawal Kumar Singh Kushwaha

**Affiliations:** National Plant Breeding and Genetics Research Centre, Nepal Agricultural Research Council, Khumaltar, Lalitpur, Nepal

Correction to: *Scientific Reports* 10.1038/s41598-022-10982-7, published online 05 May 2022

The original version of this Article contained errors in the Materials and methods section.

In the Materials and methods, under the subheading ‘Experimental methods’,

“For the grease test (trade name: MP-3 Grease, Nepal Multilube System Ind. Birganj, Nepal), each plot was demarcated diagonally, with only the plant whorl that belongs on the diagonal line included.”

now reads:

“For the grease test (trade name: MP-3 Grease, Nepal Multilube System Ind. Birganj, Nepal), each plot was demarcated diagonally, with only the plant whorl that belongs on the diagonal line plus the infested whorl per plot included.”

Under the same subheading,

“With severe fall armyworm larvae damage (50% of the total crop) on standing maize, the plots were demarcated diagonally, and the plants along the diagonal line had a small amount (0.10–0.15 g) of grease applied to the portion of the maize lower leaf that touched the soil.”

now reads:

“With severe fall armyworm larvae damage (50% of the total crop) on standing maize, the plots were demarcated diagonally, and the plants along the diagonal line along with the infested whorl per plot had a small amount (0.10-0.15g) of grease applied to the portion of the maize lower leaf that touched the soil.”

The original Article has been corrected.

